# Reducing the 10-year risk of ischemic cardiovascular disease to receive early cardiovascular benefits from bariatric surgery for obesity in China

**DOI:** 10.3389/fcvm.2022.978682

**Published:** 2022-10-11

**Authors:** Yinhui Li, Jia Liu, Biao Zhou, Xiaohui Li, Zhenyu Wu, Hua Meng, Guang Wang

**Affiliations:** ^1^Department of Endocrinology, Beijing Chao-Yang Hospital, Capital Medical University, Beijing, China; ^2^Department of General Surgery and Obesity, Metabolic Disease Center, China-Japan Friendship Hospital, Beijing, China

**Keywords:** bariatric surgery, ischemic cardiovascular disease, early cardiovascular benefits, obesity, China

## Abstract

**Background:**

Cardiovascular risk due to obesity can be improved greatly by bariatric surgery. However, there is no research involving appropriate model for evaluating cardiovascular disease risk reduction in bariatric surgery for obesity in China. We selected the ischemic cardiovascular disease (ICVD) risk score that accurately predict cardiovascular risk in Chinese adults to evaluate the 10-year risk of ICVD and estimated early cardiovascular benefits of bariatric surgery in obese Chinese patients through its reduction.

**Methods:**

From 2017 to 2019 we followed up 107 patients 6 months after surgery and measured the ICVD 10-year risk and other cardiovascular factors before and after surgery.

**Results:**

There were significant reductions in the ICVD total score (*p* < 0.001) and ICVD 10-year risk (%) (*p* < 0.001) 6 months post-operation compared with baseline. Furthermore, we found significant reductions in body mass index (BMI), body adiposity index (BAI), low-density lipoprotein (LDL), small dense-low-density lipoprotein (sd-LDL) and triglycerides (TG) 6 months after surgery compared with pre-operation (all *p* < 0.05). The decrease in ICVD total score was correlated with excess BMI loss (%EBMIL), reduced BAI, reduced LDL, reduced sd-LDL and reduced TG respectively (all *p* < 0.05) at 6 months post-operation. Moreover, there were significant reductions in the ICVD total score in the male subgroup [3 (3, 5) vs. 2.5 (2, 4), *p* < 0.001] and female subgroup [3 (2, 4) vs. 2 (1, 3), *p* < 0.001] 6 months post-operation compared with baseline. At last there were also significant reductions in the ICVD total score in the diabetic subgroup [5 (4, 6) vs. 4 (3, 5), *p* < 0.001] and non-diabetic subgroup [2 (2,3) vs. 2 (1, 2), *p* < 0.001] 6 months post-operation compared with baseline.

**Conclusions:**

Bariatric surgery could provide early cardiovascular benefits for patients with obesity in China by reducing the 10-year risk of ICVD. Both men and women with obesity achieved cardiovascular benefits according to bariatric surgery, so did diabetic and non-diabetic patients.

## Introduction

In the period between 1975 and 2016, the number of obese patients increased from 100 million to 671 million worldwide (1). With the growing global pandemic of obesity, there has also been a dramatic increase in the number of obese adults in China (2). Obesity is a public health problem associated with an increase in mortality and many comorbidities (3, 4), such as diabetes mellitus, hypertension, dyslipidaemia, and cardiovascular disease. Of these comorbidities, cardiovascular disease has the worst prognosis, and studies have found that obesity is also a major risk factor for cardiovascular disease (5). Common weight loss interventions include intensive lifestyle modification, pharmacotherapy, and bariatric surgery. To our knowledge, clinical trials have not yet shown that intensive lifestyle intervention and pharmacotherapy for obesity can decrease cardiovascular events (6). In comparison, bariatric surgery can more effectively reduce cardiovascular risk in obese patients than intensive lifestyle and medical treatment combined (7). Doumouras et al. demonstrated that bariatric surgery was associated with a lower incidence of major adverse cardiovascular events (MACE) in patients with cardiovascular disease (including ischemic heart disease and heart failure) and severe obesity (8). A meta-analysis of 39 studies suggested that bariatric surgery reduced all cause and cardiovascular mortality in patients with obesity compared with nonsurgical treatment (9). Domienik-Karłowicz et al. confirmed a significant decrease in the risk of cardiovascular diseases assessed by 2 selected risk algorithms: the Systemic Coronary Risk Evaluation (SCORE) scale and the Framingham Risk Score (FRS) in bariatric procedures. The participants and the cardiovascular risk models were from America or Europe (10). Batsis et al. proved the directionality of cardiovascular risk with estimated relative risk reduction for bariatric surgery patients using the FRS and Cardiovascular Munster Heart Study (PROCAM) risk score. In this study, the participants and cardiovascular risk models were from America or Germany (11). Although several studies have evaluated how the outcome and risk of cardiovascular disease are affected by bariatric surgery, there is no research involving an appropriate model for evaluating cardiovascular disease risk reduction in bariatric procedures for obesity in Chinese individuals. The ischemic cardiovascular disease (ICVD) risk prediction model has a satisfying predictive capability for estimating the 10-year integrated cardiovascular risk, it can accurately represent cardiovascular risk in China (12). Therefore, we used the ICVD assessment model to evaluate reductions in the 10-year risk of ICVD by bariatric surgery in obese Chinese patients. Moreover, we assessed improvements in some related cardiovascular risk factors due to bariatric surgery. In a word, we aimed to explore early cardiovascular benefits for patients with obesity in China provided by bariatric surgery.

## Methods

### Participants and design

We prospectively selected eligible obese patients to undergo bariatric surgery according to guidelines for obesity surgery (13, 14) and followed up 6 months after surgery in the general surgery department & obesity and metabolic disease Center of the China-Japan Friendship Hospital from August 2017 to September 2019. According to bariatric surgery indications, patients underwent laparoscopic sleeve gastrectomy (LSG) (15). The present research included obese Chinese participants between 35 and 59 years old who agreed to join the study and completed the follow-up. The exclusion criteria were as follows: (1) patients with secondary obesity caused by the endocrine system, such as the thalamus, pituitary, thyroid or gonadal glands; (2) patients with severe hepatic or renal insufficiency, cardiopulmonary insufficiency or other serious disease; (3) people with severe heart failure, valvular heart disease, cardiomyopathy, coronary heart disease (CHD) and stroke; (4). patients using antiplatelet aggregation therapy or statins. A total of 119 patients were enrolled in the current study. Written informed consent was given by 114 of them, but 7 patients did not come to 6-month follow-up visit after the operation. Prospective data were collected for the remaining 107 patients for analysis and risk score calculation. We confirm that the work was conducted in accordance with the Declaration of Helsinki (1964). This study was approved by the Ethics Committee of the China-Japan Friendship Hospital.

## Measurements

The tests for each participant comprised anthropometric measurements, blood sampling for biochemical measurements and ICVD risk assessment. Anthropometric indexes, including weight, height, waist circumference (WC), hip circumference (HC), systolic blood pressure (SBP), and diastolic blood pressure (DBP), were measured by well-trained investigators following a standard protocol. Height and weight were measured by a scale, with the subjects wearing light clothing and no shoes. WC was measured at the midpoint between the iliac crest and lowest rib. HC was used to measure the maximum circumference of the hip at the pubic symphysis level. Blood pressure was measured in a sitting position with a mercury sphygmomanometer. SBP and DBP were reported as the average of three repeated measurements at 30-s intervals. After a 12-h overnight fast, whole blood and serum samples were collected for each subject. Biochemical variables, including triglyceride (TG), total cholesterol (TC), high-density lipoprotein (HDL), low-density lipoprotein (LDL), and small dense-low-density lipoprotein (sd-LDL) were determined using a biochemical auto-analyzer (Hitachi 7060, Tokyo, Japan). Fasting blood glucose (FBG) was analyzed with a glucose oxidase method with the Beckman Glucose Analyzer (Beckman Instruments, Irvine, CA, USA), and fasting insulin (FINS) was analyzed by electrochemiluminescence immunoassay (Roche). Free fatty acid (FFA) was determined by using enzymatic colorimetric methods (16). Body mass index (BMI) was calculated by weight (kg)/height (m)^2^, percent excess BMI loss (%EBMIL)= (Initial BMI – Post-operative BMI)/ (Initial BMI – 25) × 100 (17), and body adiposity index (BAI)=HC (cm)/height (m)^1.5^-18 (18). Homeostasis model assessment of insulin resistance (HOMA-IR) was calculated by FBG × FINS/22.5 (19).

**Figure 1:**
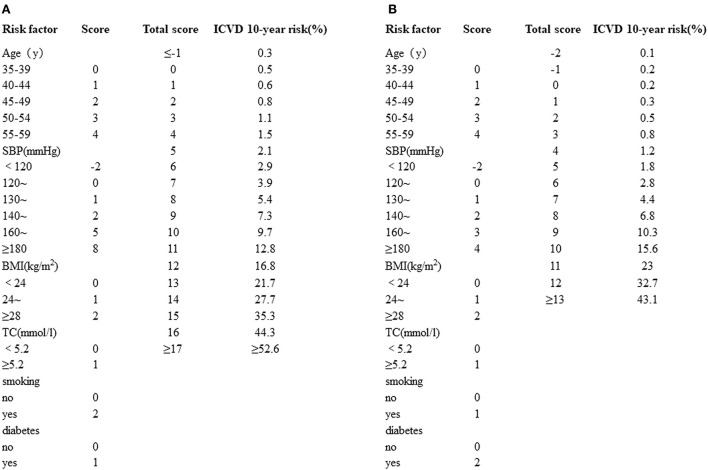
**(A)** Simplified evaluation sheet for estimating 10-year risk of ICVD incidence in men. **(B)** Simplified evaluation sheet for estimating 10-year risk of ICVD incidence in women. ICVD, ischemic cardiovascular disease; SBP, systolic blood pressure; BMI, body mass index; TC, total cholesterol.

A 10-year risk prediction model for ICVD was built to estimate cardiovascular risk. SBP (mmHg), TC (mmol/l), smoking history, diabetes mellitus history, BMI (kg/m^2^) and age (years) were taken into consideration as an integrated risk score for ICVD in the different sexes. The stratification of the integrated risk scores is as follows. (1) Age: age was classified into 5-year intervals. (2) SBP: SBP was divided into 6 levels: <120, <130, <140, <160, <180 and ≥180 mmHg. (3) TC: TC was stratified into two levels, for example: <5.2 and ≥5.2 mmol/l. (4) BMI was categorized into three groups: BMI <24, 24 ≤ BMI <28 and BMI ≥ 28 kg/m^2^. (5) Smoking history: yes or no. (6) Diabetes mellitus history: yes or no. According to the standard above, all six risk scores were added together to obtain the ICVD total score. Each ICVD total score had the corresponding ICVD 10-year risk (%) (Figure 1), which can be categorized into five levels (very low risk: 0–5%; low risk: 5–10%, middle risk 10–20%, high risk: 20–40%, very high risk: >40%) (20). All patients were evaluated twice, once at baseline and again at 6 months after bariatric surgery.

### Statistical analysis

All statistical analyses were performed using SPSS for Windows, Version 23.0 (Armonk, NY: IBM Corp) and GraphPad Prism 8.0 software (GraphPad Software Inc., CA, USA). Continuous variables were expressed as the means ± standard deviations or medians and interquartile ranges. Categorical variables were presented as numbers with percentages. The differences in ICVD total score, ICVD 10-year risk, CRP, and CK-MB between pre-operative group and post-operative group were evaluated by Wilcoxon's signed rank test, the paired sample *t*-test was used to compare pre- and post-operative levels of other relevant indicators. Independent *t*-test and the Mann-Whitney test were used to compare the indicators between the male and female subgroups, as well as the diabetic and nondiabetic subgroups. Spearman correlation analysis as a non-parametric measure was used to examine the relationships between ΔICVD total score and %EBMIL, ΔBAI, ΔLDL, Δsd-LDL, ΔTG (Δ values were obtained by subtracting the 6-month value from the pre-operative value). Statistical significance was defined as *p* < 0.05 (two-tailed).

## Results

### Baseline characteristics of participants and influence of bariatric surgery on general characteristics, risk of ICVD and other cardiovascular factors

The baseline characteristics of participants were as follows: the mean age was 36.92 ± 4.87 years, there were 68 female and 39 male participants, and the mean BMI was 39.15 ± 6.06 kg/m^2^. 7 (6.54%) patients were smokers, 39 (36.45%) patients had type 2 diabetes (T2DM), and 26 (24.3%) patients had hypertension (Table 1).

**Table 1:** Pre-operative and 6-month-post-operative demographic and clinical characteristics of participants.

**Parameter**	**Pre-op**	**6-month post-op**
	**(*n* = 107)**	**(*n* = 107)**
Age (y)	36.92 ± 4.87	36.92 ± 4.87
Sex (female)	68.00 (63.55%)	68.00 (63.55%)
BMI (kg/m^2^)	39.15 ± 6.06	28.97 ± 5.10***
%EBMIL (%)		76.06 (56.55,103.32)
WC (cm)	115.09 ± 12.02	94.36 ± 12.80***
HC (cm)	121.97 ± 13.29	104.42 ± 10.38***
BAI	37.49 ± 6.58	29.57 ± 5.88***
Smokers (n)	7.00 (6.54%)	7.00 (6.54%)
Diabetes (n)	39.00 (36.45%)	39.00 (36.45%)
Hypertension (n)	26.00 (24.30)	26.00 (24.30)
SBP (mmHg)	125.04 ± 11.32	120.17 ± 1.16***
TC (mmol/l)	4.75 ± 0.89	4.57 ± 0.89*
HDL (mmol/l)	1.05 ± 0.23	1.17 ± 0.25***
LDL (mmol/l)	3.08 ± 0.71	2.89 ± 0.68*
sd-LDL (mmol/l)	1.17 ± 0.37	0.83 ± 0.27***
TG (mmol/l)	1.66 ± 0.73	0.97 ± 0.32***
FFA (mmol/l)	0.73 ± 0.28	0.61 ± 0.28**
FBG (mmol/l)	6.72 ± 2.41	4.79 ± 0.98***
HbA1c (%)	6.26 ± 1.41	5.23 ± 0.54***
HOMA-IR	9.28 ± 6.93	2.33 ± 1.34***
CRP (mg/l)	6.00 (3.00,10.00)	4.00 (2.00,5.00)***
CK-MB (ng/ml)	1.00 (0.80,1.50)	0.70 (0.50,1.03)***
ICVD total score	3.00 (2.00,5.00)	2.00 (1.00,3.00)***
ICVD 10-year risk (%)	1.10 (0.50,1.80)	0.80 (0.50,1.10)***

Data are n (%),$\bar{x}\pm$ s, or median (interquartile range).

*p < 0.05, **p < *0.01*, ***p < 0.001 vs. pre-operation.

BMI, body mass index; %EBMIL, percent excess BMI loss; WC, waist circumference; HC, hip circumference; BAI, body adiposity index; LSG, laparoscopic sleeve gastrectomy; LRYGB, laparoscopic Roux-en-Y gastric bypass; SBP, systolic blood pressure; TC, total cholesterol; HDL, high-density lipoprotein; LDL, low-density lipoprotein; sd-LDL, small dense low-density lipoprotein; TG, triglyceride; FFA, free fatty acid; FBG, fasting blood glucose; HbA1c, glycosylated hemoglobin; HOMA-IR, homeostasis model assessment of insulin resistance; CRP, C-reactive protein; CK-MB, creatine kinase-MB; ICVD, ischemic cardiovascular disease.

**Figure 2:**
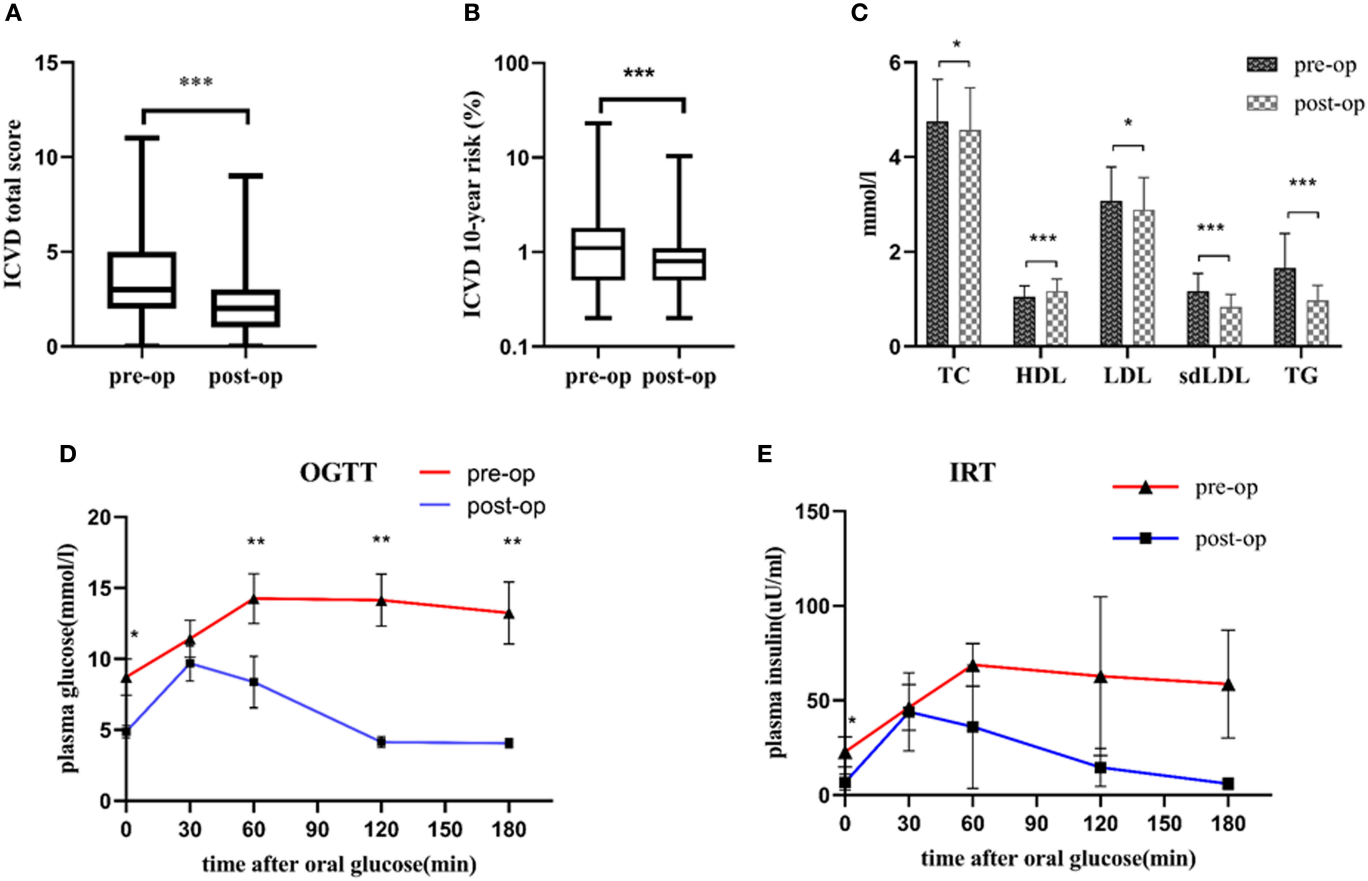
**(A)** Decreases in ICVD total score 6 months post-operation compared with pre-operation; **(B)** Decreases in ICVD 10-year risk 6 months post-operation compared with pre-operation; **(C)** Improvements in TG, TC, LDL, sd-LDL and HDL 6 months post-operation compared with pre-operation. **(D)** Decreases in the OGTT 6 months post-operation compared with pre-operation. **(E)** Decreases in IRT 6 months post-operation compared with pre-operation. *p < 0.05, **p < 0.01, ***p < 0.001 post-operation vs. pre-operation ICVD, ischemic cardiovascular disease; TG, triglyceride; TC, total cholesterol; LDL, low-density lipoprotein; sd-LDL, small dense-low-density lipoprotein; HDL, high-density lipoprotein; OGTT, oral glucose tolerance test; IRT, insulin release test. *N* = 107, continuous variables were expressed as the means ± standard deviations or medians and interquartile ranges. The differences in ICVD total score and ICVD 10-year risk between pre-operative group and post-operative group were evaluated by Wilcoxon's signed rank test. The paired-sample *t*-test was used to compare pre- and post-operative levels of TC, HDL, LDL, sdLDL, TG, OGTT and IRT.

The pre-operative and post-operative clinical characteristics of the participants are summarized in Table 1. There were significantly decreases in ICVD total score [median 3 (range 2, 5) vs. 2 (1, 3), *p* < 0.001] and ICVD 10-year risk (%) [1.1 (0.5, 1.8) vs. 0.8 (0.5, 1.1), *p* < 0.001] 6-month post-operation compared with pre-operation (Figures 2A,B). BMI, BAI, SBP, serum lipids, blood glucose, HOMA-IR, C-reactive protein (CRP), and creatine kinase-MB (CK-MB) were decreased significantly 6 months after bariatric surgery (*p* value for each trait was < 0.05). HDL increased significantly 6 months after bariatric surgery (*p* < 0.05). TG, TC, LDL, sd-LDL, and HDL are displayed in Figure 2C. A 75 g oral glucose tolerance test (OGTT) was used to evaluate glucose and insulin secretion function. Obvious improvements in glucose and insulin levels after surgery are shown in Figures 2D,E.

### The relationship between reductions in ICVD total score and cardiovascular risk factors by bariatric surgery

**Figure 3:**
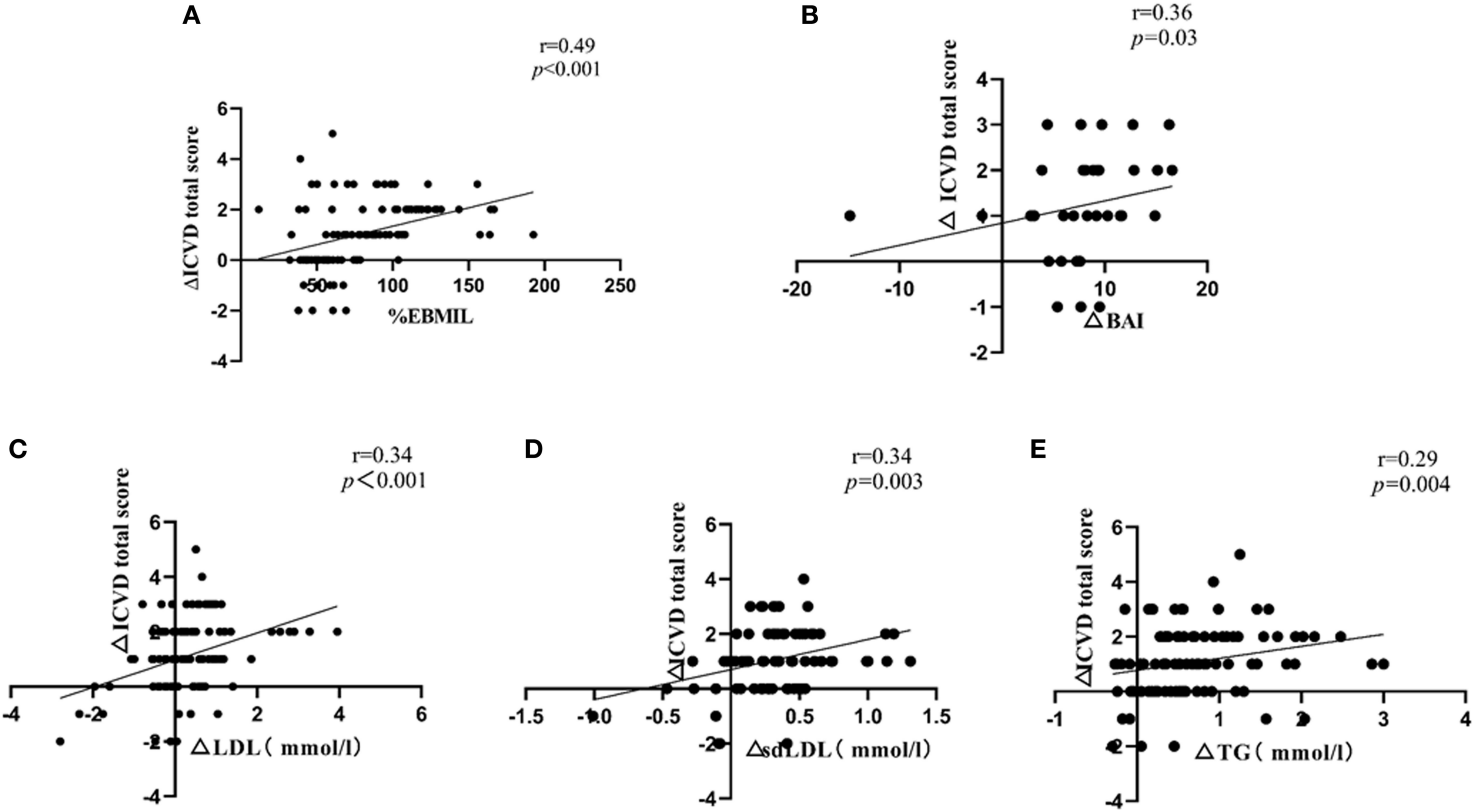
**(A)** Correlation of ΔICVD total score with %EBMIL; **(B)** Correlation of ΔICVD total score with ΔBAI; **(C)** Correlation of ΔICVD total score with ΔLDL; **(D)** Correlation of ΔICVD total score with Δsd-LDL; **(E)** Correlation of ΔICVD total score with ΔTG. Δvalues were obtained by subtracting the 6-month value from the pre-operative value; ICVD, ischemic cardiovascular disease; %EBMIL, percent excess body mass index loss; BAI, body adiposity index; LDL, low-density lipoprotein; sd-LDL, small dense-low-density lipoprotein; TG, triglyceride. *N* = 107, Spearman correlation analysis as a nonparametric measure was used to examine the relationships between ΔICVD total score and %EBMIL, ΔBAI, ΔLDL, Δsd-LDL, ΔTG.

At 6 months post-operation, correlation analysis showed a significantly positive correlation between ΔICVD (reductions in ICVD) total score and %EBMIL (r = 0.49, *p* < 0.001). The ΔICVD total score was also positively correlated with the ΔBAI (reductions in BAI) (r = 0.36, *p* = 0.03). In addition, the ΔICVD total score was positively correlated with ΔLDL (reductions in LDL) (r = 0.34, *p* < 0.001), Δsd-LDL (reductions in sd-LDL) (r = 0.34, *p* = 0.003) and ΔTG (reductions in TG) (r = 0.29, *p* = 0.004) (Figure 3).

### Reductions in ICVD total score by bariatric surgery in males and females

There were significant decreases in the ICVD total score in the male subgroup [median 3 (range 3, 5) vs. 2.5 (2, 4), *p* < 0.001] and female subgroup [median 3 (range 2, 4) vs. 2 (1, 3), *p* < 0.001] 6 months post-operation compared with pre-operation. The decreases in ICVD total score in the male subgroup were not significantly different from those in the female subgroup (Tables 2–4).

**Table 2:** Baseline and 6-month-post-operative characteristics in the male subgroup.

**Parameter**	**Baseline (*n* = 39)**	**6 months after surgery**
Age (y)	37.18 ± 4.19	37.18 ± 4.19
SBP (mmHg)	126.51 ± 12.39	120.23 ± 1.44**
BMI (kg/m^2^)	40.27 ± 6.28	30.21 ± 5.03***
TC (mmol/l)	4.73 ± 1.01	4.33 ± 0.99*
HbA1c	6.42 ± 1.59	5.22 ± 0.42***
ICVD total score	3.00 (3.00,5.00)	2.50 (2.00,4.00)***

*p < 0.05, **p < 0.01, ***p < 0.001 vs. pre-operation.

SBP, systolic blood pressure; BMI, body mass index; TC, total cholesterol; HbA1c, glycosylated hemoglobin; ICVD, ischemic cardiovascular disease.

**Table 3:** Baseline and 6-month-post-operative characteristics in the female subgroup.

**Parameter**	**Baseline (*n* = 68)**	**6 months after surgery**
Age (y)	36.76 ± 5.25	36.76 ± 5.25
SBP (mmHg)	124.19 ± 10.66	120.13 ± 0.98**
BMI (kg/m^2^)	38.50 ± 5.88	28.26 ± 5.04***
TC (mmol/l)	4.76 ± 0.84	4.64 ± 0.85
HbA1c	6.08 ± 1.12	5.28 ± 0.62***
ICVD total score	3.00 (2.00,4.00)	2.00 (1.00,3.00)***

*p < 0.05, **p < 0.01, ***p < 0.001 vs. pre-operation.

SBP, systolic blood pressure; BMI, body mass index; TC, total cholesterol; HbA1c, glycosylated hemoglobin; ICVD, ischemic cardiovascular disease.

**Table 4:** Baseline characteristics and reduced clinical characteristics 6 months after surgery: differences between the sexes.

	**Male *n* = 39**	**Female *n* = 68**	***p***
Age (y)	37.18 ± 4.19	36.76 ± 5.25	0.67
SBP (mmHg)	126.51 ± 12.39	124.19 ± 10.66	0.31
ΔSBP	0.00 (0.00,10.00)	0 (0.00,0.75)	0.35
BMI (kg/m^2^)	40.27 ± 6.28	38.50 ± 5.88	0.15
ΔBMI	10.07 ± 3.36	10.24 ± 3.49	0.81
TC (mmol/l)	4.73 ± 0.97	4.75 ± 0.84	0.86
ΔTC	0.36 (-0.25,0.80)	0.04 (-0.28,0.68)	0.24
HbA1c	6.63 ± 1.78	6.08 ± 1.11	0.09
ΔHbA1c	0.70 (0.35,1.90)	0.60 (0.30,0.90)	0.06
ICVD total score	3.00 (3.00,5.00)	3.00 (2.00,4.00)	0.11
ΔICVD total score	1.00 (0.00,2.00)	1.00 (0.00,2.00)	0.54

SBP, systolic blood pressure; ΔSBP, reduction in systolic blood pressure 6 months after surgery; BMI, body mass index; ΔBMI, reduction in body mass index 6 months after surgery; TC, total cholesterol; ΔTC, reduction in total cholesterol 6 months after surgery; HbA1c, glycosylated hemoglobin; ΔHbA1c, reduction in glycosylated hemoglobin 6 months after surgery; ICVD, ischemic cardiovascular disease; ΔICVD, reduction in ischemic cardiovascular disease 6 months after surgery.

### Reductions in ICVD total score by bariatric surgery in diabetic and non-diabetic patients

There were significant decreases in the ICVD total score in the diabetic subgroup [median 5 (range 4, 6) vs. 4 (3, 5), *p* < 0.001] and non-diabetic subgroup [median 2 (range 2, 3) vs. 2 (1, 2), *p* < 0.001] 6 months post-operation compared with pre-operation. The decreases in ICVD total score in the diabetic subgroup were not significantly different from those in the non-diabetic subgroup (Tables 5–7).

**Table 5:** Baseline and 6-month-post-operative characteristics in the diabetic subgroup.

**Parameters**	**Baseline (*n* = 39)**	**6 months after surgery**
Age (y)	39.28 ± 7.31	39.28 ± 7.31
SBP (mmHg)	127 ± 13.62	120.03 ± 0.16**
BMI (kg/m^2^)	38.72 ± 7.10	29.46 ± 5.76***
TC (mmol/l)	4.76 ± 0.98	4.64 ± 0.94
HbA1c	7.61 ± 1.69	5.51 ± 0.78***
ICVD total score	5.00 (4.00,6.00)	4.00 (3.00,5.00)***

*p < 0.05, **p < 0.01, ***p < 0.001 vs. pre-operation.

SBP, systolic blood pressure; BMI, body mass index; TC, total cholesterol; HbA1c, glycosylated hemoglobin; ICVD, ischemic cardiovascular disease.

**Table 6:** Baseline and 6-month-post-operative characteristics in the non-diabetic subgroup.

**Parameters**	**Baseline (*n* = 68)**	**6 months after surgery**
Age (y)	35.56 ± 1.48	35.56 ± 1.48
SBP (mmHg)	123.91 ± 9.69	120.25 ± 1.45**
BMI (kg/m^2^)	39.39 ± 5.42	28.69 ± 4.70***
TC (mmol/l)	4.73 ± 0.83	4.65 ± 0.85
HbA1c	5.59 ± 0.45	5.10 ± 0.29***
ICVD total score	2.00 (2.00,3.00)	2.00 (1.00,2.00)***

*p < 0.05, **p < 0.01, ***p < 0.001 vs. pre-operation.

SBP, systolic blood pressure; BMI, body mass index; TC, total cholesterol; HbA1c, glycosylated hemoglobin; ICVD, ischemic cardiovascular disease.

**Table 7:** Baseline characteristics and reduced clinical characteristics 6 months after surgery: differences between diabetic and non-diabetic patients.

	**Diabetic**	**Non-diabetic**	***p***
	***n* = 39**	***n* = 68**	
Age (y)	39.28 ± 7.31	35.56 ± 1.48	0.003
SBP (mmHg)	127.00 ± 13.62	123.91 ± 9.69	0.22
ΔSBP	0.00 (0.00,10.00)	0.00 (0.00,1.75)	0.29
BMI (kg/m^2^)	38.72 ± 7.10	39.39 ± 5.42	0.61
ΔBMI	9.26 ± 3.66	10.70 ± 3.20	0.036
TC (mmol/l)	4.76 ± 0.98	4.73 ± 0.83	0.87
ΔTC	0.53 (−0.09,1.03)	0.03 (−0.41,0.55)	0.02
HbA1c	7.53 ± 1.68	5.59 ± 0.45	0.001
ΔHbA1c	1.65 (0.87,3.05)	0.50 (0.20,0.70)	<0.001
ICVD total score	5.00 (4.00,6.00)	2.00 (2.00,3.00)	<0.001
ΔICVD total score	0.53 (−0.09,1.03)	1.00 (0.00,2.00)	0.88

SBP, systolic blood pressure; ΔSBP, reduction in systolic blood pressure 6 months after surgery; BMI, body mass index; ΔBMI, reduction in body mass index 6 months after surgery; TC, total cholesterol; ΔTC, reduction in total cholesterol 6 months after surgery; HbA1c, glycosylated hemoglobin; ΔHbA1c, reduction in glycosylated hemoglobin 6 months after surgery; ICVD, ischemic cardiovascular disease; ΔICVD, reduction in ischemic cardiovascular disease 6 months after surgery.

## Discussion

Obesity is associated with worsening physiological parameters that promote the development and progression of cardiovascular disease (21). The Framingham study showed that 23% of CHD in men and 15% of CHD in women was attributable to excess adiposity (22). Khan et al. performed a study with follow-up in 3.2 million people from 1964 to 2015 and confirmed that obesity was associated with a significantly increased risk of cardiovascular morbidity and mortality compared with normal BMI (23). In 2017, research in the Chinese community population showed that the prevalence and forecasting risk of cardiovascular disease in overweight and obese patients was higher than those in normal weight people in 10 years (24). Guidelines from heart associations suggest reducing cardiovascular risk through weight loss (25), and bariatric surgery is the most effective way to treat obesity as well as its high risk of cardiovascular disease. It establishes a pronounced state of negative energy balance by reducing gastric volume, changing the gastrointestinal endocrine and microenvironment, and improving insulin resistance to decrease cardiovascular risk factors (26).

In the present study, compared with patients' pre-operative conditions, bariatric surgery was associated with a lower risk of cardiovascular outcomes evaluated by the ICVD risk assessment model in obese Chinese patients. The result of reducing cardiovascular risk was consistent with research from other countries (27–29). Randomized trials overseas have demonstrated that bariatric procedures are more effective than the best-available intensive medical and lifestyle interventions in promoting weight loss, improving glycemic control, serum lipid levels and blood pressure (30). Improvements in these parameters after bariatric surgery result in lower cardiovascular risk scores, as measured through validated tools such as the FRS (31). In our research, the ICVD 10-year risk was reduced significantly at 6 months post-operation vs. pre-operation. There were also significant reductions in BMI, TC, and SBP at 6 months post-operation vs. pre-operation, which were important components of the ICVD risk assessment model. The improvements above due to bariatric surgery result in lower ICVD risk. The FRS has been applied worldwide, but the ICVD risk assessment model is more suitable for evaluating cardiovascular risk in Chinese individuals than the Framingham model. The FRS was derived from a predominantly Caucasian cohort and cannot be applied to non-Caucasians; it was used to predict the risk of CHD (32). In China, the incidence of CHD is approximately one-fifth to one-third that of stroke, and approximately two-thirds of strokes are ischemic (33). It is therefore more appropriate to measure and use the integrated risk of ICVD (including CHD and ischemic stroke) rather than that of CHD alone from a prevention point of view. Liu et al. found that FRS significantly overestimated the risk of CHD in the Chinese population (32). ICVD model represented cardiovascular risk very well in Chinese people. To verify ICVD model's ability to predict cardiovascular events, Wu et al. calculated the area under the curve (AUC) of this model. The results showed that the ICVD risk assessment model's AUC was higher than Framingham's AUC in both males and females (34). Moreover, the ICVD risk assessment model is more accurate in evaluating patients at low and middle cardiovascular risk than patients at high cardiovascular risk. Most participants in our study were at low cardiovascular risk (very low risk: 0–5%; low risk: 5–10%), and they had not suffered cardiovascular events, so it was appropriate in these patients to evaluate the early cardiovascular benefits of bariatric surgery by reducing the ICVD 10-year risk.

Obesity increases the risk of cardiovascular disease. The severity of obesity depends on BMI. In our study, the BMI at 6 months post-operation was reduced significantly compared with that at baseline. %EBMIL also obviously reflects the excess weight loss by bariatric surgery. This finding was consistent with the research of Domienik-Karlowicz et al. (10). In the present research, ICVD total score reduction had a positive correlation with %EBMIL 6 months after surgery. %EBMIL was the best method after bariatric surgery to report weight loss and improvement in metabolic syndrome that reduced the onset of cardiovascular events (35); it was also associated with reductions in ICVD risk and proved the early cardiovascular benefits of bariatric surgery. In addition to BMI, another method for estimating % fat was to use height and HC to calculate BAI, which reflects adiposity distribution and is associated with endothelium-dependent microvascular reactivity. Therefore, BAI may be a surrogate parameter measure of body adiposity to predict an early atherosclerotic process (36). We found that the BAI at 6 months post-operation was significantly lower than that at baseline. The BAI reduction had a positive correlation with the ICVD total score reduction 6 months after surgery. Together, these findings supported the early cardiovascular benefits of bariatric surgery. We found that LDL, sd-LDL and TG levels at 6 months post-operation were significantly lower than those at baseline. The reduction of serum lipids above can improve atherogenesis, and their reductions had a positive correlation with ICVD total score reduction 6 months after bariatric surgery. These results meant that the early benefits from bariatric surgery reducing ICVD risk were associated with improvements in LDL, sd-LDL and TG.

The sex distribution was interesting for people who underwent bariatric surgery. Women accounted for the majority of patients. Data from the 2018 International Federation for the Surgery of Obesity and Metabolic Disorder (IFSO) global registry, reported that the proportion of female patients undergoing bariatric surgery worldwide was 73.7%, whereas the proportion of male patients was small. Men with obesity were not concerned about their health as women were unless they had corresponding health problem. Overall, 92.3% of the male population belongs to the metabolically unhealthy group, indicating that metabolic abnormalities might be a major determinant for surgical treatment in men (37). Obese men were not completely aware of the advantages of bariatric surgery. When they had obesity complications, they went to the hospital and were recommended to accept bariatric surgery. However, women with obesity pay more attention to their weight and are more likely to acknowledge the benefits of bariatric surgery. Therefore, the proportion of obese females undergoing bariatric surgery is greater than that of obese males. In our research, women accounted for 63.55% of the patients, and both men and women achieved early cardiovascular benefits 6 months after bariatric surgery. There were no significant differences between them. Moreover, Doumouras et al. also reported that bariatric surgery was associated with lower cardiovascular mortality. In the subgroup analyses, bariatric surgery was associated with an equally reduced cardiovascular mortality risk for both men and women (38). The above results indicated that although a small proportion of men underwent bariatric surgery, they had the same early cardiovascular benefits as women. This benefit could encourage men with increasing obesity to undergo bariatric surgery and achieved these early cardiovascular benefits.

In this research, we found that bariatric surgery could provide cardiovascular benefits not only for obesity with diabetes, but also for obesity without diabetes. There were no significant differences between them. Another study also suggested that bariatric surgery was associated with a reduced incidence of obesity-related disorders including blood glucose disorder, hypertension, dyslipidaemia, cardiovascular mortality and the development of ischemic heart disease, in both diabetic and non-diabetic patients (39).

Atherosclerosis is an early change in cardiovascular disease. Obesity increases insulin resistance (IR), which aggravates systemic inflammation, endothelial dysfunction and dyslipidaemia and ultimately accelerates atherosclerosis (40). In addition to ICVD risk, we found that some cardiovascular indexes that participated in the formation of atherosclerosis were improved by bariatric surgery. HOMA-IR at 6 months post-operation was significantly lower than that at pre-operation. Griffo E's study also found a 50% decrease in HOMA-IR at week 2 after bariatric surgery (41). CRP at 6 months post-operation was significantly lower than that at baseline, and Yadav et al. also showed a large improvement in CRP at 6 and 12 months after bariatric surgery. We predicted that the early cardiovascular benefits following bariatric surgery might be attributed to improvements in HOMA-IR, a reduction in CRP to relieve the inflammatory response, and suppression of the formation of atherosclerosis. SBP at 6 months post-operation was significantly lower than that at baseline, and a similar change was found in Yadav et al.'s research (42). We speculate that bariatric surgery decreased HOMA-IR, reduced hypertension, relieved the pressure on the blood vessel endothelium, and prevented atherogenesis. Furthermore, we still found that FFA, TG, LDL, and sd-LDL were reduced significantly at 6 months post-operation vs. baseline, and HDL was increased significantly at 6 months post-operation vs. baseline. The above changes were similar to those in previous bariatric surgery studies (10, 11, 42). In summary, we presumed that bariatric surgery improved insulin resistance, decreased the influx of FFAs from adipose tissue to the liver, reduced triglyceride synthesis, and affected the transportation of lipids, leading to an increase in HDL and a decrease in LDL and sd-LDL, ultimately decreased the atherogenic dyslipidaemia complex (43). Therefore, bariatric surgery might bring early cardiovascular benefits by preventing the formation of atherosclerosis. At the end of our study, there was no cardiovascular event.

The main limitations of this study were that the population assessed was from a single center, the sample was relatively small, and the follow-up was short. Further studies should enlarge the sample size and increase the follow-up time to provide more comprehensive clinical evidence of the early cardiovascular benefits of bariatric surgery.

## Conclusion

Our study confirmed early cardiovascular benefits in bariatric procedures in obese Chinese patients. To the best of our knowledge, for the first time, we found that bariatric surgery could reduce the ICVD 10-year risk in Chinese obese patients. Both men and women with obesity reaped cardiovascular benefits 6 months after bariatric surgery, as did diabetic and non-diabetic patients.

## Data availability statement

The raw data supporting the conclusions of this article will be made available by the authors, without undue reservation.

## Ethics statement

The studies involving human participants were reviewed and approved by Ethics Committee of the China-Japan Friendship Hospital. The patients/participants provided their written informed consent to participate in this study.

## Author contributions

YL, HM, and GW designed the study, analyzed the data, and wrote the manuscript. JL and BZ collected data and revised the manuscript for intellectual content. XL and ZW contributed to the database and helped with data statistics and interpretation. GW and HM are the guarantors of this work and are accountable for the integrity of the data and the accuracy of the data analysis. All authors read and approved the final version of the manuscript.

## Funding

This study was supported by grants from the National Natural Science Foundation of China (Nos. 81770792, 81972137) to GW. The Foundation of Beijing Municipal Science & Technology Commission (No. Z151100004015065) to HM.

## Conflict of interest

The authors declare that the research was conducted in the absence of any commercial or financial relationships that could be construed as a potential conflict of interest.

## Publisher's note

All claims expressed in this article are solely those of the authors and do not necessarily represent those of their affiliated organizations, or those of the publisher, the editors and the reviewers. Any product that may be evaluated in this article, or claim that may be made by its manufacturer, is not guaranteed or endorsed by the publisher.

## References

[1] Worldwide Trends in B. Overweight, and obesity from 1975 to 2016: a pooled analysis of 2416 population-based measurement studies in 128·9 Million children, adolescents, and adults. Lancet. (2017) 390:2627–42. 10.1016/s0140-6736(17)32129-3 29029897 PMC5735219

[2] Hu L Huang X You C Li J Hong K Li P. Prevalence of overweight, obesity, abdominal obesity and obesity-related risk factors in Southern China. PLoS ONE. (2017) 12:e0183934. 10.1371/journal.pone.0183934 28910301 PMC5598943

[3] Finucane MM Stevens GA Cowan MJ Danaei G Lin JK Paciorek CJ. National, regional, and global trends in body-mass index since 1980: systematic analysis of health examination surveys and epidemiological studies with 960 country-years and 9.1 million participants. Lancet. (2011) 377:557–67. 10.1016/S0140-6736(10)62037-5 21295846 PMC4472365

[4] Garvey WT Mechanick JI Brett EM Garber AJ Hurley DL Jastreboff AM. American association of clinical endocrinologists and American college of endocrinology comprehensive clinical practice guidelines for medical care of patients with obesity. Endocr Pract. (2016) 22 Suppl 3:1–203. 10.4158/EP161365.GL 27219496

[5] Mi YJ Zhang B Wang HJ Yan J Han W Zhao J. Prevalence and secular trends in obesity among Chinese adults, 1991–2011. Am J Prev Med. (2015) 49:661–9. 10.1016/j.amepre.2015.05.005 26275960 PMC4615397

[6] Srivastava G Apovian CM. Current pharmacotherapy for obesity. Nat Rev Endocrinol. (2018) 14:12–24. 10.1038/nrendo.2017.122 29027993

[7] Adams TD Arterburn DE Nathan DM Eckel RH. Clinical outcomes of metabolic surgery: microvascular and macrovascular complications. Diabetes Care. (2016) 39:912–23. 10.2337/dc16-0157 27222549 PMC5562446

[8] Doumouras AG Wong JA Paterson JM Lee Y Sivapathasundaram B Tarride JE. bariatric surgery and cardiovascular outcomes in patients with obesity and cardiovascular disease: a population-based retrospective cohort study. Circulation. (2021) 143:1468–80. 10.1161/CIRCULATIONAHA.120.052386 33813836

[9] van Veldhuisen SL Gorter TM van Woerden G de Boer RA Rienstra M Hazebroek EJ. Bariatric surgery and cardiovascular disease: a systematic review and meta-analysis. Eur Heart J. (2022) 43:1955–69. 10.1093/eurheartj/ehac071 35243488 PMC9123239

[10] Domienik-Karłowicz J Ziemiański P Małkowski P Kosieradzki M Pruszczyk P Lisik W. Retrospective study of 6-month reduction in risk of developing cardiovascular diseases and type 2 diabetes mellitus in severely obese patients over 60 years of age following bariatric surgery. Med Sci Monit. (2019) 25:2577–82. 10.12659/MSM.915937 30958811 PMC6467173

[11] Batsis JA Sarr MG Collazo-Clavell ML Thomas RJ Romero-Corral A Somers VK. Cardiovascular risk after bariatric surgery for obesity. Am J Cardiol. (2008) 102:930–7. 10.1016/j.amjcard.2008.05.040 18805125 PMC2773706

[12] Wu Y Liu X Li X Li Y Zhao L Chen Z. Estimation of 10-year risk of fatal and non-fatal ischemic cardiovascular diseases in Chinese adults. Circulation. (2006) 114(21):2217–25. 10.1161/CIRCULATIONAHA.105.607499 17088464

[13] Mechanick JI Youdim A Jones DB Garvey WT Hurley DL McMahon MM. Clinical practice guidelines for the perioperative nutritional, metabolic, and nonsurgical support of the bariatric surgery patient−2013 update: cosponsored by American association of clinical endocrinologists, the obesity society, and american society for metabolic and bariatric surgery. Obesity. (2013) 21 Suppl 1:S1–27. 10.1002/oby.20461 23529939 PMC4142593

[14] Aminian A Chang J Brethauer SA Kim JJ. Asmbs Updated position statement on bariatric surgery in class I obesity (BMI 30–35 kg/m^2^). Surg Obes Relat Dis. (2018) 14:1071–87. 10.1016/j.soard.2018.05.025 30061070

[15] Sztuczka E Zukowska W Jackowski M Janik MR Paśnik K Michalik M. Recommendations for the standards of equipping of the bariatric and metabolic surgery center. Pol Przegl Chir. (2018) 90:52–6. 10.5604/01.3001.0012.0977 30426939

[16] Zheng R Yang M Bao Y Li H Shan Z Zhang B. Prevalence and determinants of metabolic health in subjects with obesity in Chinese population. Int J Environ Res Public Health. (2015) 12:13662–77. 10.3390/ijerph121113662 26516886 PMC4661606

[17] Brethauer SA Kim J El Chaar M Papasavas P Eisenberg D Rogers A. Standardized outcomes reporting in metabolic and bariatric surgery. Obes Surg. (2015) 25:587–606. 10.1007/s11695-015-1645-3 25802064

[18] Bergman RN Stefanovski D Buchanan TA Sumner AE Reynolds JC Sebring NG. A better index of body adiposity. Obesity. (2011) 19:1083–9. 10.1038/oby.2011.38 21372804 PMC3275633

[19] Lyssenko V Almgren P Anevski D Perfekt R Lahti K Nissén M. Predictors of and longitudinal changes in insulin sensitivity and secretion preceding onset of type 2 diabetes. Diabetes. (2005) 54:166–74. 10.2337/diabetes.54.1.166 15616025

[20] Wu YF Zhou BF Gao RL Li Y Zhao LC Yang J. A study on evaluation of the risk of ischemic cardiovascular disease in Chinese and the development of simplifiedtols for evalation. Chin J Cardiol. (2003) 31:893–901. 10.3760/j:issn:0253-3758.2003.12.005

[21] Lavie CJ Milani RV Ventura HO. Adipose composition and heart failure prognosis: paradox or not? J Am Coll Cardiol. (2017) 70:2750–1. 10.1016/j.jacc.2017.10.017 29191322

[22] Wilson PW D'Agostino RB Sullivan L Parise H Kannel WB. Overweight and obesity as determinants of cardiovascular risk: the framingham experience. Arch Intern Med. (2002) 162:1867–72. 10.1001/archinte.162.16.1867 12196085

[23] Khan SS Ning H Wilkins JT Allen N Carnethon M Berry JD. Association of body mass index with lifetime risk of cardiovascular disease and compression of morbidity. JAMA Cardiol. (2018) 3:280–7. 10.1001/jamacardio.2018.0022 29490333 PMC5875319

[24] Chen L. Associations between obesity status and cardiovascular disease risk in chinese community population. China Medical Abstracts. (2017) 34:155.

[25] Eckel RH Jakicic JM Ard JD de Jesus JM Miller NH Hubbard VS. 2013 AHA/ACC guideline on lifestyle management to reduce cardiovascular risk: a report of the american college of cardiology/American heart association task force on practice guidelines. Circulation. (2014) 129(25_suppl_2):S76–99. 10.1161/01.cir.0000437740.48606.d1 24222015

[26] Jansen PL van Werven J Aarts E Berends F Janssen I Stoker J. Alterations of hormonally active fibroblast growth factors after Roux-en-Y gastric bypass surgery. Dig Dis. (2011) 29:48–51. 10.1159/000324128 21691104

[27] Pontiroli AE Pizzocri P Librenti MC Vedani P Marchi M Cucchi E. Laparoscopic adjustable gastric banding for the treatment of morbid (Grade 3) obesity and its metabolic complications: a 3-year study. J Clin Endocrinol Metab. (2002) 87:3555–61. 10.1210/jcem.87.8.8708 12161474

[28] Busetto L Sergi G Enzi G Segato G De Marchi F Foletto M. Short-term effects of weight loss on the cardiovascular risk factors in morbidly obese patients. Obes Res. (2004) 12:1256–63. 10.1038/oby.2004.159 15340108

[29] Stoopen-Margain E Fajardo R España N Gamino R González-Barranco J Herrera MF. Laparoscopic Roux-en-Y Gastric bypass for morbid obesity: results of our learning curve in 100 consecutive patients. Obes Surg. (2004) 14:201–5. 10.1381/096089204322857564 15018748

[30] Ikramuddin S Korner J Lee WJ Connett JE Inabnet WB Billington CJ. Roux-en-Y gastric bypass vs. intensive medical management for the control of type 2 diabetes, hypertension, and hyperlipidemia: the diabetes surgery study randomized clinical trial. Jama. (2013) 309:2240–9. 10.1001/jama.2013.5835 23736733 PMC3954742

[31] Arterburn D Schauer DP Wise RE Gersin KS Fischer DR Selwyn CA Jr. Change in predicted 10-year cardiovascular risk following laparoscopic Roux-en-Y gastric bypass surgery. Obes Surg. (2009) 19:184–9. 10.1007/s11695-008-9534-7 18704607

[32] Liu J Hong Y D'Agostino RB Sr Wu Z Wang W Sun J. predictive value for the chinese population of the framingham CHD risk assessment tool compared with the chinese multi-provincial cohort study. Jama. (2004) 291:2591–9. 10.1001/jama.291.21.2591 15173150

[33] Zhang LF Yang J Hong Z Yuan GG Zhou BF Zhao LC. Proportion of different subtypes of stroke in China. Stroke. (2003) 34:2091–6. 10.1161/01.STR.0000087149.42294.8C 12907817

[34] Wilson PW D'Agostino RB Levy D Belanger AM Silbershatz H Kannel WB. Prediction of coronary heart disease using risk factor categories. Circulation. (1998) 97:1837–47. 10.1161/01.CIR.97.18.1837 9603539

[35] Rossi M Serpa Neto A Rossi FM Amarante RD Alcântara GC Jr da Silva RB. Percentage of excess BMI lost correlates better with improvement of metabolic syndrome after Roux-en-Y gastric bypass in morbidly obese subjects: anthropometric indexes and gastric bypass. Surg Obes Relat Dis. (2009) 5:11–8. 10.1016/j.soard.2008.08.002 18996755

[36] Dias IB Panazzolo DG Marques MF Paredes BD Souza MG Manhanini DP. Relationships between emerging cardiovascular risk factors, Z-BMI, waist circumference and body adiposity index (Bai) on adolescents. Clin Endocrinol. (2013) 79:667–74. 10.1111/cen.12195 23469930

[37] Li M Liu Y Jin L Zeng N Wang L Zhao K. Metabolic features of individuals with obesity referred for bariatric and metabolic surgery: a cohort study. Obes Surg. (2019) 29:3966–77. 10.1007/s11695-019-04067-0 31468305

[38] Doumouras AG Hong D Lee Y Tarride JE Paterson JM Anvari M. Association between bariatric surgery and all-cause mortality: a population-based matched cohort study in a universal health care system. Ann Intern Med. (2020) 173:694–703. 10.7326/M19-3925 32805135

[39] Wiggins T Guidozzi N Welbourn R Ahmed AR Markar SR. Association of bariatric surgery with all-cause mortality and incidence of obesity-related disease at a population level: a systematic review and meta-analysis. PLoS Med. (2020) 17:e1003206. 10.1371/journal.pmed.1003206 32722673 PMC7386646

[40] Haffner SM Stern MP Hazuda HP Mitchell BD Patterson JK. Cardiovascular risk factors in confirmed prediabetic individuals. Does the clock for coronary heart disease start ticking before the onset of clinical diabetes? Jama. (1990) 263:2893–8. 10.1001/jama.263.21.2893 2338751

[41] Griffo E Nosso G Lupoli R Cotugno M Saldalamacchia G Vitolo G. Early Improvement of Postprandial Lipemia after Bariatric Surgery in Obese Type 2 Diabetic Patients. Obes Surg. (2014) 24:765–70. 10.1007/s11695-013-1148-z 24374941

[42] Yadav R Hama S Liu Y Siahmansur T Schofield J Syed AA. Effect of Roux-en-Y bariatric surgery on lipoproteins, insulin resistance, and systemic and vascular inflammation in obesity and diabetes. Front Immunol. (2017) 8:1512. 10.3389/fimmu.2017.01512 29187850 PMC5694757

[43] Rader DJ. Effect of insulin resistance, dyslipidemia, and intra-abdominal adiposity on the development of cardiovascular disease and diabetes mellitus. Am J Med. (2007) 120(3 Suppl 1):S12–8. 10.1016/j.amjmed.2007.01.003 17320517

